# Regression of Triple-Negative Breast Cancer in a Patient-Derived Xenograft Mouse Model by Monoclonal Antibodies against IL-12 p40 Monomer

**DOI:** 10.3390/cells11020259

**Published:** 2022-01-13

**Authors:** Madhuchhanda Kundu, Sumita Raha, Avik Roy, Kalipada Pahan

**Affiliations:** 1 Department of Neurological Sciences, Rush University Medical Center, Chicago, IL 60612, USA; madhuchhanda.kundu@gmail.com (M.K.); sumita_raha@rush.edu (S.R.); avik61332@gmail.com (A.R.); 2 Division of Research and Development, Jesse Brown Veterans Affairs Medical Center, 820 South Damen Avenue, Chicago, IL 60612, USA

**Keywords:** triple-negative breast cancer, patient-derived xenograft mouse model, immunotherapy, IL-12p40 monomer, tumor-associated macrophage

## Abstract

Although some therapies are available for regular breast cancers, there are very few options for triple-negative breast cancer (TNBC). Here, we demonstrated that serum level of IL-12p40 monomer (p40) was much higher in breast cancer patients than healthy controls. On the other hand, levels of IL-12, IL-23 and p40 homodimer (p40_2_) were lower in serum of breast cancer patients as compared to healthy controls. Similarly, human TNBC cells produced greater level of p40 than p40_2_. The level of p40 was also larger than p40_2_ in serum of a patient-derived xenograft (PDX) mouse model. Accordingly, neutralization of p40 by p40 mAb induced death of human TNBC cells and tumor shrinkage in PDX mice. While investigating the mechanism, we found that neutralization of p40 led to upregulation of human CD4^+^IFNγ^+^ and CD8^+^IFNγ^+^ T cell populations, thereby increasing the level of human IFNγ and decreasing the level of human IL-10 in PDX mice. Finally, we demonstrated the infiltration of human cytotoxic T cells, switching of tumor-associated macrophage M2 (TAM2) to TAM1 and suppression of transforming growth factor β (TGFβ) in tumor tissues of p40 mAb-treated PDX mice. Our studies identify a possible new immunotherapy for TNBC in which p40 mAb inhibits tumor growth in PDX mice.

## 1. Introduction

Breast cancer is the most common female cancer worldwide and the second most common cause of cancer-related deaths in women in USA [1]. Triple-negative breast cancer (TNBC) is a subtype of breast cancer that is characterized by the deficiency of estrogen receptor (ER), progesterone receptor, and human epidermal growth factor receptor 2 (HER2) [2,3]. TNBC affecting particularly younger women with a poor prognosis accounts for about 15–20% of all breast cancer. While tremendous advances have been made in other subtypes of breast cancer such as HER2^+^ tumors with the development of targeted agents against the HER2 receptor and ER^+^ breast cancers with agents to block estrogen signaling, there are no approved targeted treatments for TNBC. Historically, cytotoxic chemotherapy has been the only viable systemic treatment option for patients with TNBC.

IL-12 family of cytokines has four different members including p40 monomer (p40), p40 homodimer (p40_2_), IL-12 (p40:p35), and IL-23 (p40:p19) [4,5,6,7,8,9,10,11]. Recently we have delineated that p40 is different from other IL-12 family members to selectively inhibit IL-12Rβ1 internalization and suppress autoimmune demyelination [5]. We have also seen that certain cancer cells stave off their death with the help from p40 and that selective depletion of p40 by a functional blocking mAb leads to regression of prostate tumor in mice [4]. Here, we demonstrated that the level of p40 was significantly higher in serum of breast cancer patients than that of age-matched healthy controls. Accordingly, human TNBC cells also produced higher levels of p40 than p40_2_ and neutralization of p40 by p40 mAb induced death of TNBC cells. Finally, we delineated that p40 mAb treatment stimulated tumor-associated M1 macrophages, decreased tumor-associated M2 macrophages, and suppressed the TGFβ axis, leading to the regression of TNBC tumor in a PDX mouse model. These studies identify an anti-TNBC property of p40 mAb, which may be beneficial for TNBC patients.

## 2. Materials and Methods

### 2.1. Reagents

Human TNBC cell lines (BT-549 and HCC70) were purchased from ATCC. Cell culture materials (RPMI 1640, DMEM, L-glutamine, antibiotic/antimycotic) were purchased from Life Technologies (Carlsbad, CA, USA). Hamster IgG (cat# IR-HT-GF) was obtained from Innovative Research (Novi, MI, USA). MTT assay kit (cat# CGD1), and LDH assay kit (cat# TOX7) were purchased from Sigma (St. Louis, MO, USA). TUNEL assay kit (cat# QIA39) was purchased from Calbiochem (San Diego, CA, USA) and Annexin V assay kit (cat# K101-25) was purchased from Biovision (Milpitas, CA, USA). We purchased antibody against inducible nitric oxide synthase (iNOS) from BD Bioscience (Franklin Lakes, NJ, USA). Antibodies against ionized calcium binding adaptor molecule 1 (Iba1), PD-1, and TGFβ were purchased from Abcam (Cambridge, UK). Antibody against arginase 1 was purchased from Thermo Fisher (Waltham, MA, USA).

### 2.2. Serum Samples of Breast Cancer Patients

Serum samples of breast cancer patients prior to treatment and age-matched healthy controls were obtained from Discovery Life Sciences, Los Osos, CA, USA.

### 2.3. Animals

Animal maintaining and experiments were in accordance with National Institute of Health guidelines and were approved by the Institutional Animal Care and Use committee of the Rush University of Medical Center, Chicago, IL. Patient-derived xenograft (PDX) model (ID# TM00096) was purchased from Jackson Laboratory, Bar Harbor, ME, USA. In this model, TNBC tumor fragments at passage P1-P9 (invasive ductal carcinoma; TNBC ER^−^PR^−^HER2^−^) were engrafted in the flank of female 6–8 week old NOD scid gamma (NSG) mice. Mice were shipped to us by Jackson Laboratory within ~2 weeks following tumor engraftment. PDX mice were maintained in our temperature-controlled animal vivarium with adequate food and water.

### 2.4. Tumor Measurement

Tumor growth was measured with a caliper and tumor cross-sectional area was determined with the formula (mm^2^ = longest diameter X shortest diameter). Treatment with p40 mAb started when the tumor sizes reached 0.6–0.8 cm^2^ in area. The p40 mAb a3-3a was injected once a week intraperitoneally in 0.1 ml volume of sterile PBS-1% normal mouse serum. The tumors were then measured to determine progression or regression. Infrared dye (Alexa 800-conjugated 2DG dye; Licor) was injected via tail-vein on the day before imaging analysis. Mice were sacrificed at the end of the study and tumor tissues were collected for different biochemical analyses.

### 2.5. Sandwich ELISA

Sandwich ELISA was used to quantify p40_2_ and p40 as described by us [4,5,12,13]. Briefly, for p40_2_, mAb a3-1d (1.3 mg/mL) was diluted 1:3000 and added to each well (100 µL/well) of a 96-well ELISA plate for coating. The biotinylated p40_2_ mAb d7-12c (2 mg/mL) was diluted 1:3000 and used as detection antibody. Similarly for p40, mAb a3-3a (1.3 mg/mL) and biotinylated p40 mAb a3-7g (2 mg/mL) were also diluted 1:3000 and used as coating and detection antibodies, respectively [12]. Concentrations of IFN-γ, IL-12, IL-23, and IL-10 were measured in serum or tissue homogenates by ELISA (eBioscience/ThermoFisher (San Diego, CA, USA)), according to the manufacturer’s instructions as described before [5]. 

#### 2.5.1. Isolation of Splenocytes

Spleens isolated from treated or untreated PDX mice were placed into a cell strainer and mashed with a syringe plunger. Resulting single-cell suspensions were treated with RBC lysis buffer (Sigma-Aldrich), washed, and cultured in RPMI 1640 supplemented with 10% FBS, 50 μM 2-ME, 2 mM L-glutamine, 100 U/mL penicillin, and 100 μg/mL streptomycin as described [14,15].

#### 2.5.2. Flow Cytometry

Single-cell suspensions isolated from mouse spleen or tumor were stained with Zombie Aqua™ Fixable Viability Kit (Biolegend, San Diego, CA, USA) according to the manufacturer’s instructions. Cells were washed with FACS buffer (ThermoFisher) and stained with FITC-anti-human CD4 antibody and APC/Cy7-anti-human CD8 antibody (Biolegend) for extracellular stains. For IFNγ staining, cells were stained with PE-anti-human IFNγ antibody (Biolegend) and detected by flow cytometry analysis. Only PE-treated and unstained cells served as control. Flow cytometric analyses were performed using the LSRFortessa analyzer (BD Biosciences) and analyzed using the FlowJo Software (v10) as described [4,5].

#### 2.5.3. Tissue Preparation and Immunohistochemistry

Paraffin embedded tissue sections were prepared and tissue sections were cut 5 micron in size as described [13,16,17]. To eliminate endogenous peroxidase activity, tissue sections were deparaffinized, rehydrated and incubated with 3% H_2_O_2_ in methanol for 15 min at room temperature. Antigen retrieval was performed at 95 °C for 20 min by placing the slides in 0.01 M sodium citrate buffer (pH 6.0). After blocking, the slides were then incubated with the primary antibodies for 2 h at room temperature followed by washing and incubation with Cy2 or Cy5 (Jackson ImmunoResearch Laboratories, West Grove, PA, USA) secondary antibodies at room temperature for 1 h [18,19].

### 2.6. Cell Viability Measurements

#### 2.6.1. MTT Assay

Mitochondrial activity was measured with the 3-(4, 5-dimethylthiazol-2-yl)-2, 5-diphenyltetrazolium bromide (MTT) assay (Sigma) as described before [17,20]. The cells were grown on 24-well culture plates with 500 μL of medium and treated with various reagents according to the experimental design. At the end of the treatment period, 300 μL of culture medium were removed from each well, and 20 μL of MTT solution (5 mg/mL) were added and incubated for 1 h.

#### 2.6.2. LDH Assay

The activity of lactate dehydrogenase (LDH) was measured using the direct spectrophotometric assay using an assay kit from Sigma as described before [17,20].

#### 2.6.3. Liver Toxicity Assay

The activity of alanine aminotransferase (ALT) or serum glutamic-pyruvic transaminase (SGPT) was monitored in serum using an assay kit from Sigma following manufacturer’s protocol.

#### 2.6.4. TUNEL and Actin Double-Labeling

Following treatments with p40 mAb, TUNEL assays were performed as described earlier [21,22]. Briefly, tumor tissue sections were blocked using blocking buffer followed by treatment with 20 μg/mL proteinase K at room temperature and one wash with PBS. Next, the samples were incubated for 90 min in terminal deoxynucleotidyl transferase (TdT) equilibration buffer containing anti-Actin antibody. After three washes in PBST, the sections were incubated in fluorescein-fragEL TdT reaction mix containing TdT enzyme and secondary antibody for 60 min at 37 °C. Prior to mounting, the samples were washed twice in PBS. Finally, the samples were mounted using mounting media containing 4,6,-DiAmidino-2-PhenylIndole (DAPI), which allows the visualization of total cell population and observed for fluorescein-labeled DNA fragments.

### 2.7. Real-Time PCR

Total RNA was isolated from tumor tissues using Ultraspec II RNA Reagent (Biotecx Laboratories Inc.) according to the manufacturer’s protocol [23,24,25].

To remove any contaminating genomic DNA, total RNA was digested with DNase. Then DNase-digested RNA was analyzed by real-time PCR using the primers (Table 1) in the ABI-Prism7700 Sequence Detection System (Applied Biosystems) as described previously [4,5].

### 2.8. Statistical Analysis

For tumor regression, quantitative data were presented as the mean ± SEM. Statistical significance were accessed via one-way ANOVA with Student-Newman-Keuls posthoc analysis. Other data were expressed as means ± SD of three independent experiments. Statistical differences between means were calculated by the Student’s *t*-test. A *p*-value of less than 0.05 (*p* < 0.05) was considered statistically significant.

## 3. Results

### 3.1. Levels of IL-12, IL-23, p40_2_, and p40 in Serum of Breast Cancer Patients

**Figure 1:**
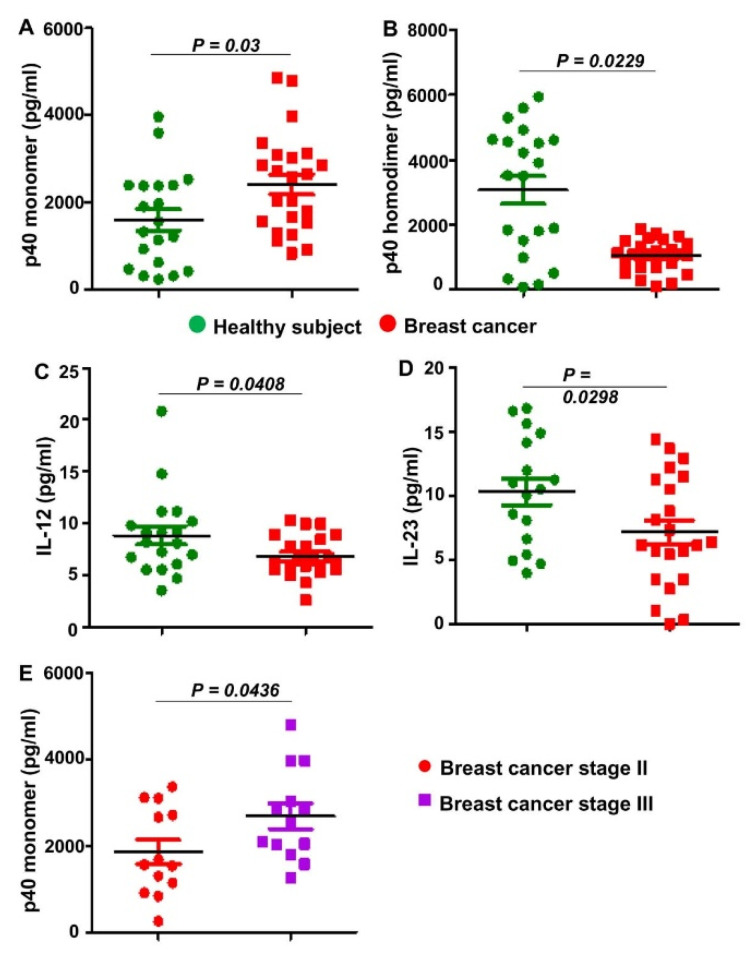
Levels of IL-12 family of cytokines in serum of breast cancer patients. Serum of drug-naïve breast cancer patients (*n* = 23) and age-matched healthy controls (*n* = 21) obtained from Discovery Life Sciences (Los Osos, CA) were analyzed for p40 (**A**), p40_2_ (**B**), IL-12 (**C**), and IL-23 (**D**) by sandwich ELISA. Serum of stage II and stage III breast cancer patients (*n* = 13) were also analyzed for p40 (**E**). Results were statistically analyzed by Student’s *t*-test.

Recently we have seen that the level of p40 is much higher in the serum of prostate cancer patients as compared to healthy controls [4]. To understand if this observation is specific to prostate cancer or not, we also measured levels of IL-12, IL-23, p40_2_, and p40 in serum of breast cancer patients (*n* = 12) and age-matched healthy controls (*n* = 12). Since disease modifying therapies may alter the levels of these cytokines, we used serum of only pretreated breast cancer patients. Similar to that observed in prostate cancer patients, the level of p40 was significantly higher in serum of breast cancer patients as compared to healthy controls (Figure 1A). In contrast, levels of p40_2_ (Figure 1B), IL-12 (Figure 1C) and IL-23 (Figure 1D) were significantly lower in breast cancer cases relative to healthy controls, signifying the specificity of the finding. We also measured the level of p40 in serum of stage II and stage III breast cancer patients and found significantly higher level of p40 in serum of stage III patients than stage II patients (Figure 1E), indicating cancer stage-specific increase in serum level of p40.

### 3.2. Immunotherapy with Monoclonal Antibody against IL-12 p40 Monomer (p40 mAb) Stimulates Death in Human TNBC Cells

**Figure 2:**
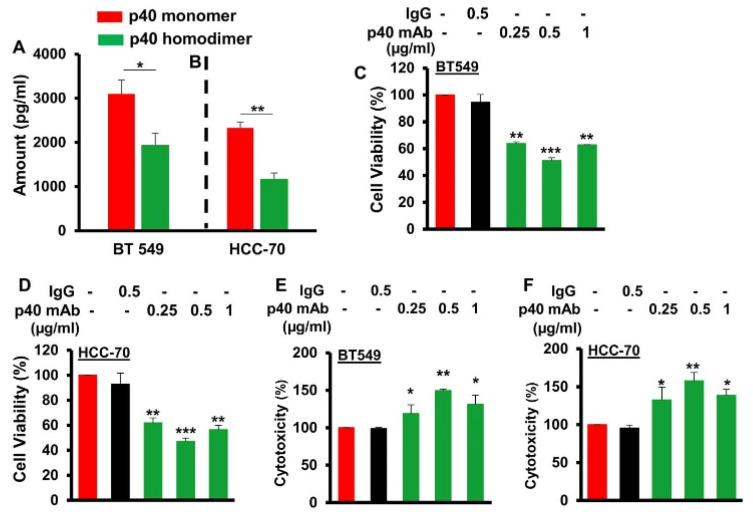
Monoclonal antibody-mediated neutralization of p40 induces death of different human TNBC cells. Levels of p40 and p40_2_ were measured in supernatants of human TNBC cells (**A**, BT-549; **B**, HCC70) by ELISA. Results are mean ± SD of three different experiments. * p < 0.05; ** p < 0.01. BT-549 (**C**,**E**) and HCC70 (**D**,**F**) cells were treated with p40 mAb for 24 h under serum-free condition followed by monitoring MTT metabolism (**C**,**D**) and LDH release (**E**,**F**). Results are mean ± SD of three separate experiments. * p < 0.05; ** p < 0.01; *** p < 0.001.

Since it was difficult to get serum samples of pre-treated TNBC cases in sufficient numbers, next, we monitored levels of p40 and p40_2_ in human TNBC cell lines. Human TNBC cells (BT-549 and HCC70) were cultured under serum-free condition for 48 h followed by measuring the levels of p40 and p40_2_ in supernatants by sandwich ELISA. Similar to that found in serum of breast cancer cases, the level of p40 was significantly higher than p40_2_ in supernatants of both BT-549 (Figure 2A) and HCC70 (Figure 2B) cells. Therefore, we examined the role of p40 in the survival human TNBC cells using p40 mAb a3-3a. Earlier we demonstrated that p40 mAb a3-3a neutralized the production of nitric oxide and tumor necrosis factor α (TNFα) in peritoneal macrophages induced by only p40, but not p40_2_, IL-12 and IL-23 [12]. Recently, we have also seen that single administration of p40 mAb a3-3a, but not control IgG, stimulates clinical symptoms of experimental allergic encephalomyelitis (EAE), an animal model of multiple sclerosis (MS) [5]. This is in sharp contrast to the functions of mAb against p40_2_, IL-12, and IL-23 in EAE [13,26,27], signifying that function of p40 is different from other IL-12 family members (p40_2_, IL-12, and IL-23). Here, we noticed that the p40 mAb a3-3a decreased MTT (Figure 2C,D) and increased LDH release (Figure 2E,F) in both BT-549 (Figure 2C,E) and HCC70 (Figure 2D,F) cells, indicating induction of cell death in TNBC cells by neutralization of p40. This result was specific as control hamster IgG had no effect on either LDH or MTT in human TNBC cells.

### 3.3. Immunotherapy with p40 mAb Induces Regression of Tumor in Patient-Derived Xenograft (PDX) Mouse Model of TNBC

In the absence of any effective genetically engineered mouse model for TNBC, PDX models are widely used to evaluate preclinical assessment of any new therapeutic approaches. We used the PDX model (ID# TM00096; Jackson Lab) for this study. In this model, TNBC tumor (invasive ductal carcinoma) were engrafted in the flank of female NOD scid gamma (NSG) mice.

**Figure 3:**
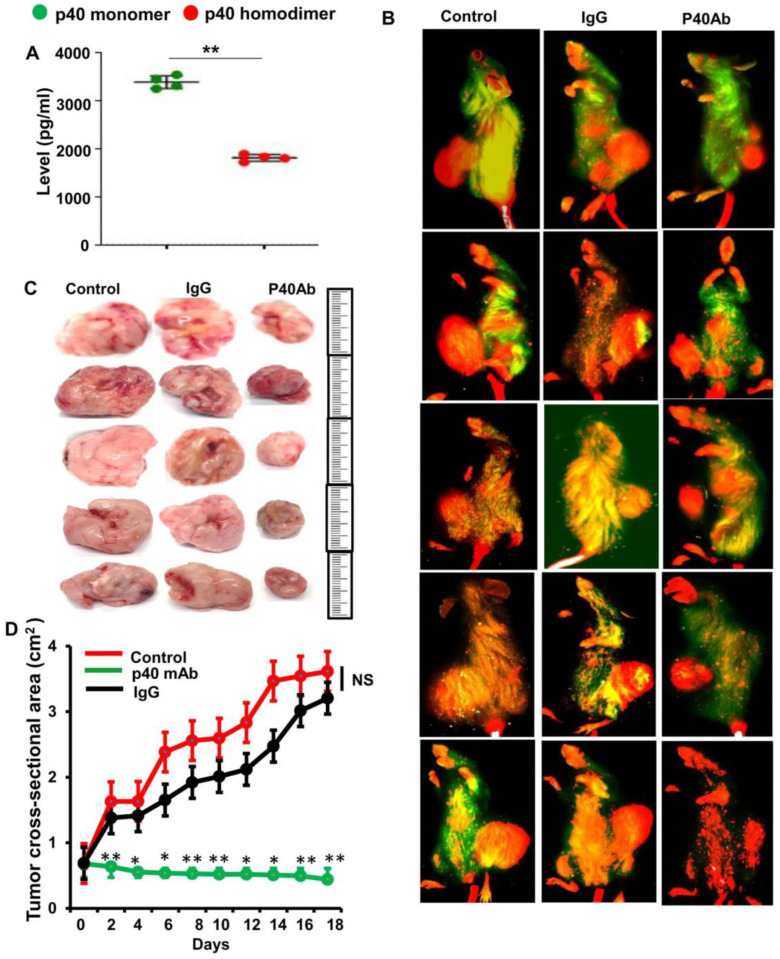
Regression of TNBC tumor in vivo in patient-derived xenograft (PDX) mice by p40 mAb. (**A**) Female 6–8 week old NOD scid gamma (NSG) mice were engrafted TNBC tumor fragments at passage P1-P9 (invasive ductal carcinoma; TNBC ER^−/−^/PR^−/−^/HER2^−/−^) in the flank. After 6 weeks of engraftment, levels of p40 and p40_2_ were measured in serum by sandwich ELISA. Results are mean ± SEM of four mice (*n* = 4) per group. ** *p* < 0.01. (**B**) After about 4 weeks of engraftment, when tumors of PDX mice (*n* = 5 per group) were 0.6–0.8 cm^2^ in size, mice were treated with p40 mAb (right panel) and hamster IgG (middle panel) at a dose of 2 mg/kg body wt once a week. After 2 weeks, tumors were labeled with Alexa800 conjugated 2DG dye via tail vein injection and then imaged in Licor Odyssey infrared imaging system. Results were compared with control group (Left panel). (**C**) Tumors were excised from the flank of all groups of mice. Five mice (*n* = 5) were included in each group. (**D**) Tumor size was monitored every alternate day. Results are mean ± SEM of five different mice.

At first, we measured the level of p40 in serum of PDX mice and found greater level of p40 than p40_2_ in serum about 4 weeks after TNBC tumor engraftment (Figure 3A). Therefore, to examine the role of p40 in the progression of TNBC tumor, we examined the effect of p40 mAb on tumor size and the death of tumor tissue in PDX mice. When the tumor had reached 0.6–0.8 cm^2^ in area, mice were treated with p40 mAb a3-3a at a dose of 2 mg/kg body/week intraperitoneally (i.p.) for 2 weeks.

The tumor size was recorded every alternate day. After 2 weeks of treatment, tumors were labeled with IR dye 800-conjugated 2-deoxy-d-glucose via tail vein injection and then imaged in a LI-COR Odyssey infrared scanner. Interestingly, we observed that administration of p40 mAb significantly reduced the size of tumors as evident from whole-animal IR images (Figure 3B) and images of excised tumors (Figure 3C). It was clear from the tumor regression curve that the size of tumors in the p40 mAb-treated group was much less than the control group (Figure 3D). In contrast, control hamster IgG had no such effect (Figure 3B–D).

### 3.4. Immunotherapy with p40 mAb Stimulates the Death Response in Tumor Tissues of PDX Mouse Model of TNBC

**Figure 4:**
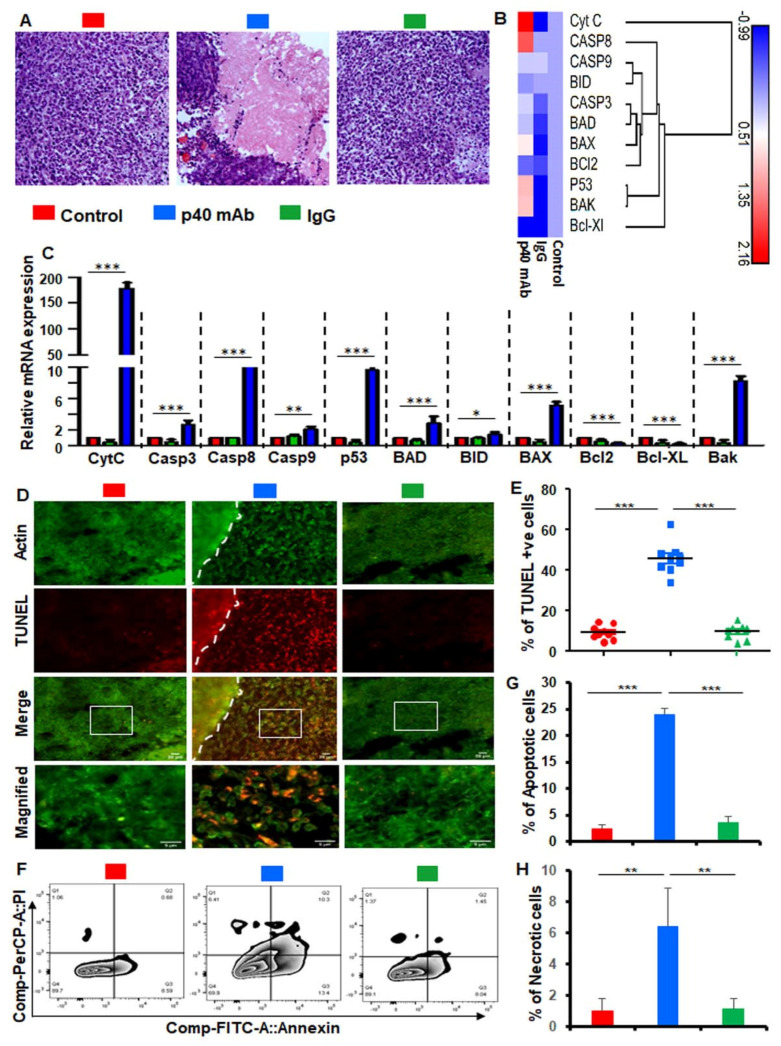
Stimulation of death response in TNBC tumor of PDX mice by p40 mAb. Female 6–8 week old NSG mice were engrafted TNBC tumor in the flank. After about 4 weeks of engraftment, when tumors of PDX mice (*n* = 5 per group) were 0.6–0.8 cm^2^ in size, mice were treated with p40 mAb and hamster IgG at a dose of 2 mg/kg body wt once a week. After 2 weeks of treatment, H&E staining (**A**) was performed on tumor sections. Tumor tissues were analyzed for the expression of different death-related genes by a custom mRNA array (**B**), which was then plotted with heat map explorer software. (**C**) Real-time mRNA analyses of 11 different death-related genes. Results are mean ± SEM of five mice. *** p < 0.001. Tumor tissue sections were double-labeled for actin and TUNEL (**D**) followed by counting of TUNEL-positive cells in two sections of each of five mice per group (**E**). Single-cell suspensions isolated from tumor tissues were studied by dual FACS for propidium iodide (PI) and Annexin V (**F**) in the LSRFortessa analyzer (BD Biosciences) followed by analysis using the FlowJo Software (v10). Quantification of apoptotic (PI^−^Annexin V^+^ early apoptotic + PI^+^Annexin V^+^ late apoptotic; **G**) and necrotic (Annexin V^−^PI^+^; **H**) cells. Results are mean ± SEM of three PDX mice per group. * *p* < 0.05; ** p < 0.01; *** p < 0.001.

Evasion of apoptosis, the cell’s natural mechanism for programed cell death, is one of the hallmarks of cancer cells. Therefore, next, we monitored apoptosis or death response in these tumor tissues. Although we did not see complete regression of TNBC tumors by p40 mAb, H&E staining showed almost empty core as we did not find the presence of any live cells in the core of the tumor of p40 mAb-treated PDX mice in comparison to either control untreated or IgG-treated PDX mice (Figure 4A). To understand the cell death process, we checked the mRNA expression of different genes related to cell death and survival in treated and untreated tumor tissues using a custom gene array.

Gene array (Figure 4B) followed by real-time PCR analysis of individual genes (Figure 4C) clearly showed that p40 mAb treatment markedly increased the expression of apoptosis-related genes such as cytochrome C, caspase 3, caspase 8, caspase 9, p53, BAD, BID, BAX, and BAK in tumor tissues of PDX mice. On the other hand, we observed decrease in survival-associated genes such as Bcl_2_ and Bcl-XL in tumor tissues of p40 mAb-treated PDX mice (Figure 4B,C). Our TUNEL results also clearly showed that the population of TUNEL-positive cells in the p40 mAb-treated tumors was higher than either control or IgG-treated tumors (Figure 4D,E). To confirm this observation further, we performed dual FACS analysis with propidium iodide and annexin V (Figure 4F) and found that p40 mAb treatment markedly increased the level of both apoptotic (Figure 4G) and necrotic (Figure 4H) cells in tumor tissues of PDX mice. Together, these results suggest that neutralization of p40 by p40 mAb is capable of inducing cell death in TNBC tumors.

### 3.5. Upregulation of Human CD4^+^IFNγ^+^ and CD8^+^IFNγ^+^ T Cells In Vivo in Spleen of PDX Mice after p40 mAb Treatment

Cancer cells do not routinely die off like other cells in the human body as cancer cells are known to escape death due to a change in immune surveillance. Fortunately, we have been endowed with CD8^+^IFNγ^+^ and CD4^+^IFNγ^+^ T cells to deal with such a situation. While collaborations between CD8^+^IFNγ^+^ and CD4^+^IFNγ^+^ T cells are needed to kill tumor cells [28,29,30], several studies have reported that both of these cell types are suppressed in cancer patients during disease progression [31].

**Figure 5:**
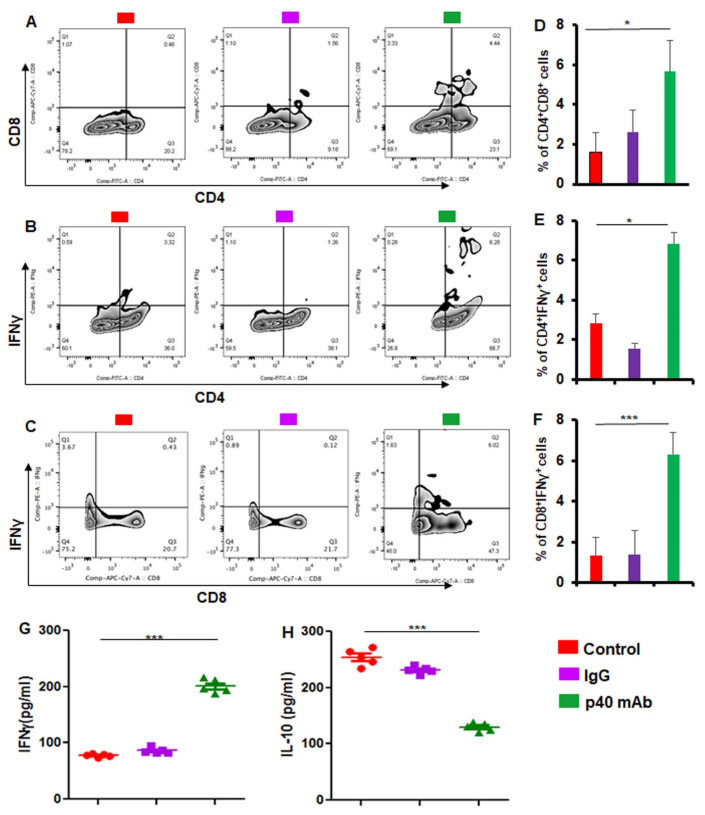
Stimulation of adaptive immune response in spleen of PDX mice by p40 mAb treatment. Female 6–8 week old NSG mice were engrafted TNBC tumor in the flank. After about 4 weeks of engraftment, when tumors of PDX mice were 0.6-0.8 cm^2^ in size, mice were treated with p40 mAb and hamster IgG at a dose of 2 mg/kg body wt once a week. After 2 weeks of treatment, spleens were harvested and levels of human CD4^+^CD8^+^ (**A**), CD4^+^IFNγ^+^ (**B**) and CD8^+^IFNγ^+^ (**C**) T cells were monitored in splenocytes by FACS using a BD LSRFortessa™ cell analyzer. Percentages of human CD4^+^CD8^+^ (**D**), CD4^+^IFNγ^+^ (**E**) and CD8^+^IFNγ^+^ (**F**) T cells were calculated. Results are mean ± SEM of five mice (*n* = 5) per group (one analysis per mouse). Levels of human IFNγ (**G**) and IL-10 (**H**) were measured in serum of all groups (*n* = 5) of mice by sandwich ELISA. * *p* < 0.05; *** *p* < 0.001.

Although we employed PDX model in NSG mice, a number of studies have demonstrated the development of functional T cells in humanized mouse models [32,33]. CD3^+^ and CD8^+^ T cells are readily identified in blood, spleen, and bone marrow of a PDX model of TNBC in NSG mice [34]. According to Najima et al. [35], human CD8^+^ T cells in NSG mice also recognize the tumor associated antigen, Wilms tumor 1 (WT1), suggesting that human mature T cells recognizing specific antigens can be generated in the humanized mouse model. Therefore, we monitored the effect of p40 mAb treatment on human CD8^+^IFNγ^+^ and CD4^+^IFNγ^+^ T cells in treated and untreated PDX mice. Interestingly, we found that p40 mAb treatment markedly increased the overall adaptive immune response as monitored by an increase in human CD4^+^, CD8^+^ as well as CD4^+^CD8^+^ T cells in splenocytes of p40 mAb-treated PDX mice in comparison to either untreated or control IgG-treated PDX mice (Figure 5A,D). Furthermore, double labeling of splenocytes for CD4 and IFNγ revealed an upregulation of human CD4^+^IFNγ^+^ immune response in PDX mice by p40 mAb treatment (Figure 5B,E). This result was specific as we did not see any increase in human CD4^+^IFNγ^+^ T cells by control IgG treatment (Figure 5B,E).

Similarly, when we labeled splenocytes for CD8 and IFNγ, we found marked increase in human CD8^+^IFNγ^+^ T cells in TNBC mice by treatment with p40 mAb, but not IgG (Figure 5C,F). Our ELISA results from serum of PDX mice also demonstrate marked increase in human IFNγ in serum of PDX mice after treatment with p40 mAb, but not control IgG (Figure 5G). In contrast, we found significant decrease in human IL-10 in serum of p40 mAb-treated PDX mice (Figure 5H), highlighting the specificity of our finding that neutralization of p40 by mAb in PDX mice selectively upregulates CD8^+^IFNγ^+^ and CD4^+^IFNγ^+^ T cell responses.

### 3.6. Immunotherapy with p40 mAb Increases Human CD4^+^IFNγ^+^ and CD8^+^IFNγ^+^ Immune Responses in Tumor Tissues of PDX Mouse Model of TNBC

**Figure 6:**
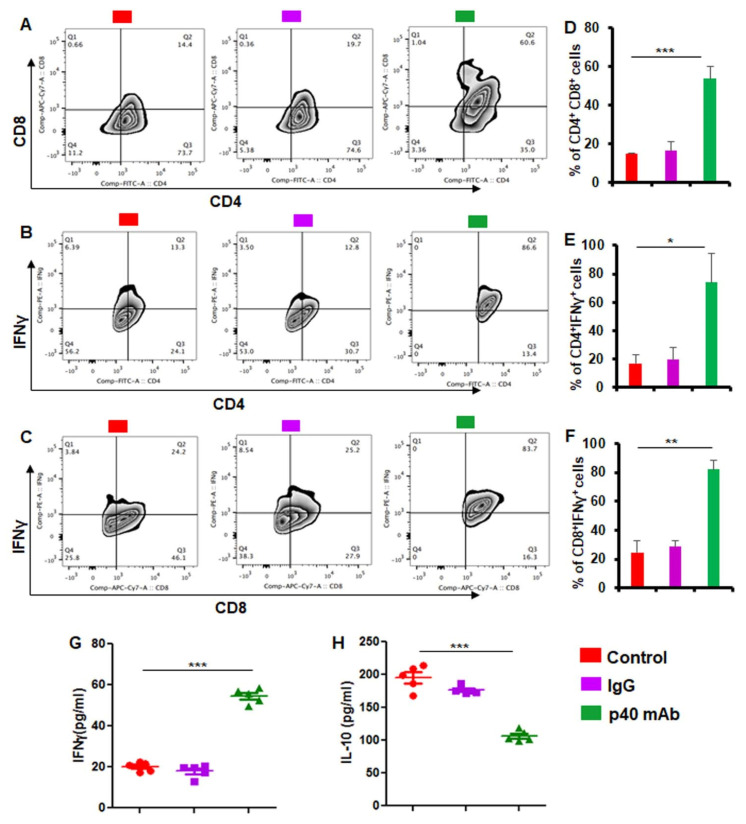
Neutralization of p40 by p40 mAb stimulates adaptive immune response in TNBC tumor of PDX mice. Female 6–8 week old NSG mice were engrafted TNBC tumor in the flank. After about 4 weeks of engraftment, when tumors of PDX mice were 0.6-0.8 cm^2^ in size, mice were treated with p40 mAb and hamster IgG at a dose of 2 mg/kg body wt once a week. After 2 weeks of treatment, tumor tissues were harvested and levels of human CD4^+^CD8^+^ (**A**), CD4^+^IFNγ^+^ (**B**) and CD8^+^IFNγ^+^ (**C**) T cells were monitored in single cell suspensions by FACS using a BD LSRFortessa™ cell analyzer. Percentages of human CD4^+^CD8^+^ (**D**), CD4^+^IFNγ^+^ (**E**) and CD8^+^IFNγ^+^ (**F**) T cells were calculated. Results are mean ± SEM of five mice (*n* = 5) per group (one analysis per mouse). Levels of human IFNγ (**G**) and IL-10 (**H**) were measured in TNBC homogenates of all groups (*n* = 5) of mice by sandwich ELISA. * *p* < 0.05; ** *p* < 0.01; *** *p* < 0.001.

**Figure 7:**
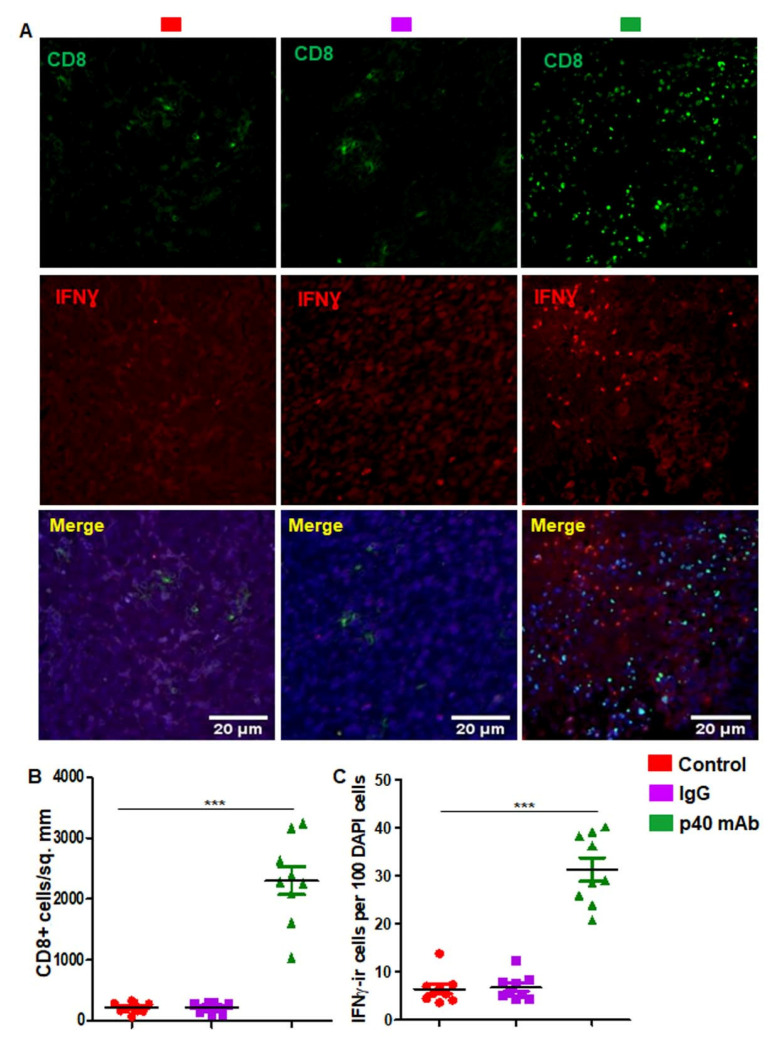
Neutralization of p40 by p40 mAb induces the infiltration of human CD8^+^IFNγ^+^ T cells into TNBC tumor of PDX mice. Female 6–8 week old NSG mice were engrafted TNBC tumor in the flank. After about 4 weeks of engraftment, when tumors of PDX mice were 0.6–0.8 cm^2^ in size, mice were treated with p40 mAb and hamster IgG at a dose of 2 mg/kg body wt once a week. After 2 weeks of treatment, tumor sections were double-labeled for human CD8 and human IFNγ (**A**) followed by counting of CD8^+^ (**B**) and IFNγ^+^ (**C**) cells in two sections of each of five mice per group. *** *p* < 0.001.

Since p40 mAb upregulated human CD4^+^IFNγ^+^ and CD8^+^IFNγ^+^ T cells in the spleen of PDX mice, next we investigated whether p40 mAb treatment was capable of mounting these immune responses in vivo in tumor tissues. Similar to that observed in the spleen, p40 mAb treatment also upregulated human CD4^+^CD8^+^ (Figure 6A,D), CD4^+^IFNγ^+^ (Figure 6B,E) and CD8^+^IFNγ^+^ T cells (Figure 6C,F) in tumor tissues of PDX mice. Since the infiltration of CD8^+^IFNγ^+^ T cells into the tumor is key to tumor regression, next, we monitored the infiltration of these cells into TNBC tumor in different groups of PDX mice. Double-labeling of tumor cross sections with antibodies against human CD8 and human IFNγ (Figure 7A) followed by counting of CD8^+^ (Figure 7B) and IFNγ^+^ (Figure 7C) T cells clearly show marked infiltration of human CD8^+^ cells capable of releasing IFNγ into the tumor of PDX mice after treatment with p40 mAb, but not control IgG.

### 3.7. The p40 mAb Treatment Upregulates M1 Macrophages while Suppressing M2 Ones in the Tumor Tissues of PDX Mouse Model of TNBC

**Figure 8:**
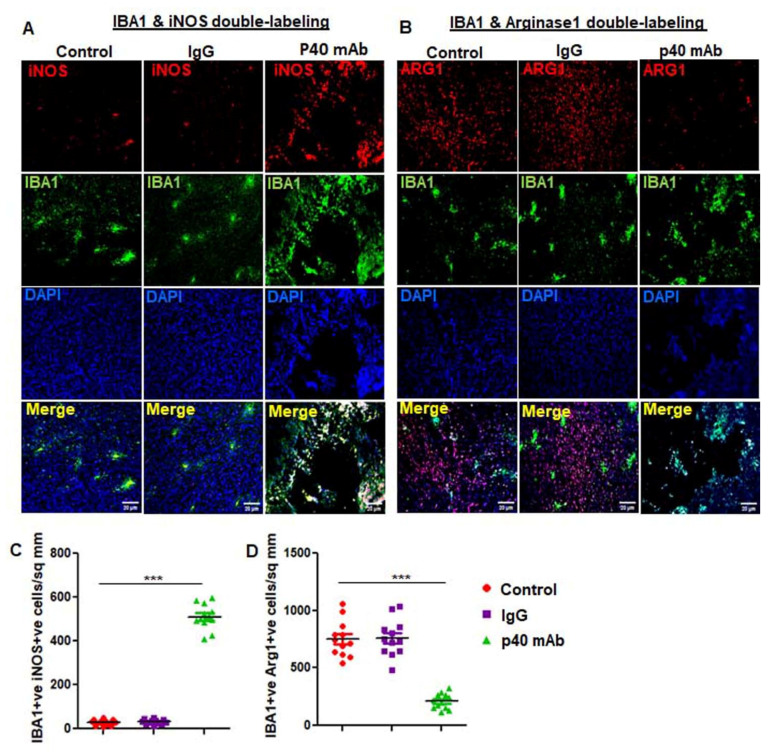
Neutralization of p40 by p40 mAb downregulates tumor-associated M2 (TAM2) macrophages, while upregulating TAM1 macrophages in TNBC tumor of PDX mice. Female 6–8 week old NSG mice were engrafted TNBC tumor in the flank. After about 4 weeks of engraftment, when tumors of PDX mice were 0.6–0.8 cm^2^ in size, mice were treated with p40 mAb and hamster IgG at a dose of 2 mg/kg body wt once a week. After 2 weeks of treatment, tumor cross sections were double-immunolabeled for either Iba1 & iNOS (**A**) or Iba1 & Arg1 (**B**). DAPI was used to stain nuclei. Cells positive for Iba1 & iNOS (**C**) and Iba1 & Arg1 (**D**) were counted in one section (2–3 images per section) of each of five different mice per group. *** *p* < 0.001.

Tumor associated macrophages (TAMs) play a critical role in the pathogenesis of various cancers including TNBC [36]. TAMs usually polarize to either M1 exhibiting anti-tumor activity or M2 with tumor promoting functions. While TAM1s express high amounts of inducible nitric oxide synthase (iNOS), TAM2s are characterized by arginase 1 (ARG1) [37]. Therefore, we examined the status of TAM1/TAM2 in TNBC tumor after p40 mAb treatment. As evident from Figure 8A,C, the number of iNOS^+^Iba1^+^ TAM1s that was low in control TNBC tumor dramatically increased after treatment with p40 mAb, but not control IgG.

In contrast, Arg1^+^Iba1^+^ TAM2s were downregulated in TNBC tumor after treatment with p40 mAb, but not control IgG (Figure 8B,D), suggesting that p40 mAb immunotherapy is capable of switching TAM2 to TAM1 in TNBC tumor.

**Figure 9:**
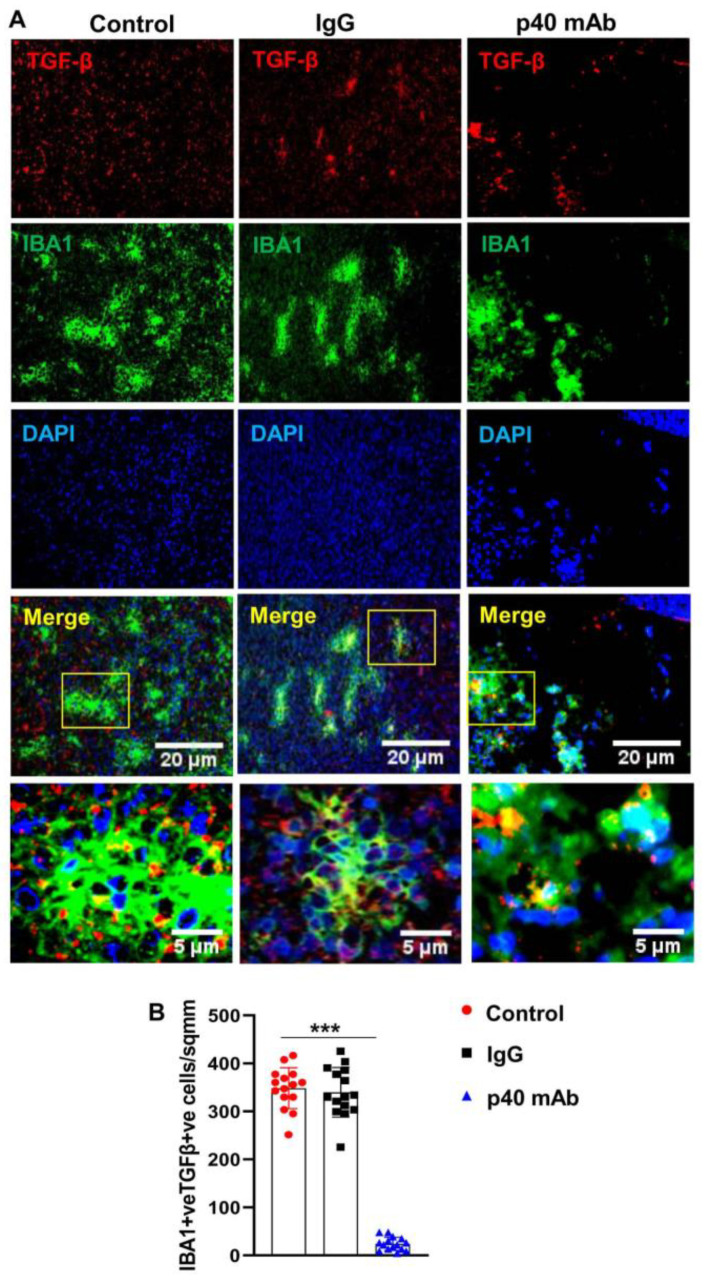
Neutralization of p40 by p40 mAb decreases the level of TGFβin TNBC tumor of PDX mice. Female 6–8 week old NSG mice were engrafted TNBC tumor in the flank. After about 4 weeks of engraftment, when tumors of PDX mice were 0.6–0.8 cm^2^ in size, mice were treated with p40 mAb and hamster IgG at a dose of 2 mg/kg body wt once a week. After 2 weeks of treatment, tumor cross sections were double-immunolabeled for Iba1 and TGFβ (**A**). DAPI was used to stain nuclei. Cells positive for Iba1 and TGFβ (**B**) were counted in one section (2–3 images per section) of each of five different mice per group. * *p* < 0.05; *** *p* < 0.001.

Although TGFβ has a paradoxical role in cancer, in the later stage, it plays an important role in tumor progression allowing cancer cells to escape immune surveillance [38]. Accordingly, we found the presence of TGFβ in Iba1-positive macrophages as well as other cells of TNBC tumor (Figure 9A,B). However, treatment of PDX mice with p40 mAb, but not control IgG, led to strong inhibition of TGFβ in TNBC tumor (Figure 9A,B). These results suggest that in addition to switching TAM2 to TAM1, neutralization of p40 by p40 mAb is also capable of dismantling other suppressor circuits in the tumor microenvironment.

### 3.8. The p40 mAb Immunotherapy Is Not Toxic in PDX Mouse Model of TNBC

**Figure 10:**
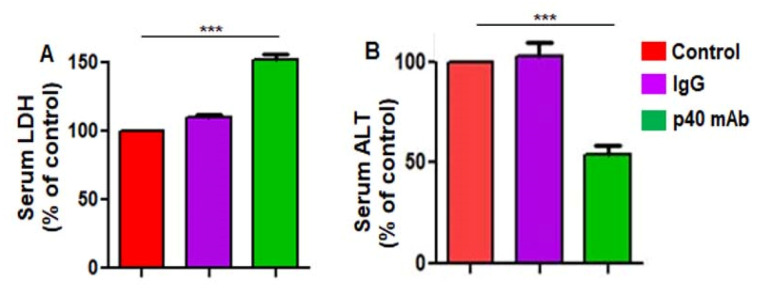
Neutralization of p40 by p40 mAb is not toxic for PDX mice. Female 6–8 week old NSG mice were engrafted TNBC tumor in the flank. After about 4 weeks of engraftment, when tumors of PDX mice were 0.6–0.8 cm^2^ in size, mice were treated with p40 mAb and hamster IgG at a dose of 2 mg/kg body wt once a week. After 2 weeks of treatment, the level of LDH (**A**) and ALT (**B**) was assayed in serum using assay kits (Sigma). Results are mean ± SEM of five mice per group. *** *p* < 0.001.

Alanine aminotransferase (ALT) is probably the most widely used clinical biomarker of liver well-being. Similarly, higher serum LDH than normal levels usually indicates tissue damage. Therefore, to understand whether p40 mAb elicited any toxic effects, we measured LDH and ALT in serum of all groups of mice. While p40 mAb treatment increased the level of serum LDH in PDX mice (Figure 10A) due to the death of TNBC tumor, the level of serum ALT was markedly inhibited in p40 mAb-treated PDX mice (Figure 10B), indicating that p40 mAb immunotherapy is not toxic in TNBC mice and that p40 mAb treatment reduces liver toxicity in TNBC mice. These results were specific as control IgG did not alter the level of either LDH or ALT in serum of PDX mice.

## 4. Discussion

TNBC is an aggressive type of breast cancer associated with limited treatment options and as a result, TNBC accounts for 5% of all cancer-related deaths annually. With current therapies, the median overall survival for TNBC is 10.2 months with a 5-year survival rate of ~65% for local tumors and 11% for those with tumor spreading to distant organs. Since TNBC is chemotherapy sensitive, chemotherapy is the standard of care, especially in cases where surgery is not an option. Recently, a number of immunotherapies in combination with different investigational drugs are also being tested for TNBC [39,40]. IL-12 is an important cytokine for eliciting cell-mediated immune response [6]. Antigen-presenting cells upon activation through Toll-like receptors and/or interactions with CD4^+^ T cells produce this heterodimeric (p35:p40) cytokine [6,41].

Recently, a number of immunotherapies in combination with different investigational drugs are also being tested for TNBC [39,40]. IL-12 is an important cytokine for eliciting cell-mediated immune response [6]. Antigen-presenting cells upon activation through Toll-like receptors and/or interactions with CD4^+^ T cells produce this heterodimeric (p35:p40) cytokine [6,41]. Although IL-12 p40 monomer (p40) was known as biologically inactive, recently we have seen greater levels of p40 in serum of prostate cancer patients as compared to healthy controls and regression of prostate tumor in mice after neutralization of p40 by a specific mAb [4]. Interestingly, we have seen opposite results in serum of multiple sclerosis (MS) patients in which the level of p40 in lower in serum of MS patients as compared to healthy controls and supplementation of p40 inhibits autoimmune demyelination in mice [5]. Here, we demonstrate that the level of p40 is significantly higher in serum of breast cancer patients as compared to age-matched healthy controls and that human TNBC cells as well as PDX mouse model of TNBC also produce excess p40. Accordingly, mAb-mediated neutralization of p40 induces death in human TNBC cells and suppresses tumor growth in a PDX mouse model of TNBC. These results indicate the possible immunotherapeutic prospect of p40 mAb in TNBC.

To achieve tumor regression, it is almost mandatory to induce apoptosis and/or necrosis in tumor tissues. From several angles, we have demonstrated greater death response in TNBC tumor after p40 mAb treatment.

Our conclusion is dependent on the following observations: First, H&E staining showed an empty or a dead tumor core in p40 mAb-treated PDX mice. On the other hand, we did not notice such hollow core in tumors of either untreated or control IgG-treated PDX mice. Second, as expected, we found marked upregulation of apoptosis-related genes such as cytochrome C, caspase 3, caspase 8, caspase 9, p53, BAD, BID, BAX, and BAK in tumor tissues of p40 mAb-treated PDX mice as compared to that of either untreated or control IgG-treated PDX mice. Third, number of TUNEL-positive cells was much higher in tumors of p40 mAb-treated PDX mice than that of either untreated or control IgG-treated PDX mice. Fourth, dual FACS staining with PI and annexin V revealed increase in early apoptotic (PI negative and annexin V positive), late apoptotic (PI positive and annexin V positive) as well as necrotic (PI positive and annexin V negative) cells in tumors of p40 mAb-treated PDX mice as compared to untreated or control IgG-treated PDX mice. Therefore, p40 mAb may be considered for inducing cell death response in TNBC tumors.

Upregulation of CD8^+^ cytotoxic-1 T (Tc1) lymphocyte-mediated adaptive immune response is one of the important mechanisms for the induction of death response in different cancers including TNBC. Accordingly, Tc1 cells capable of producing IFNγ are a major immunological effector cell population mediating resistance to cancer [42,43]. Tc1 cells can also eradicate the growth and metastasis of malignant tumor cells [43]. However, in clinical trials, only a limited number of patients respond to Tc1 cell therapy [44]. Although underlying mechanisms are unknown, it is probably due to lack of T cell helper arm [29,30]. Similar to Tc1 cells, CD4^+^ T helper 1 (Th1) cells that are essential for generating cellular immunity do not directly kill tumor cells. However, Th1 cells play an important role in priming Tc1-mediated antitumor responses [29]. It is possible that Th1 cells provide IL-2 needed to elicit Tc1-mediated anti-tumor immunity [28]. It has been also reported that Th1 cells play an important role in the induction of Tc1 cell responses through the activation of DC via CD40 ligation [45]. Moreover, Th1 cells are required in defining the magnitude and persistence of Tc1 responses and for Tc1 infiltration into tumors [46,47]. Therefore, upregulation of both Tc1 and Th1 responses together is important for successful cancer immunotherapy. It is nice to see that p40 mAb treatment markedly upregulates both human CD4^+^IFNγ^+^ and CD8^+^IFNγ^+^ T cell responses in spleen and TNBC tumors of PDX mice.

Mechanisms by which neutralization of p40 upregulates IFNγ is also becoming clear. IFNγ production is usually driven by IL-12 [6,8]. While the heterodimeric cytokine IL-12 signals via a heterodimer of IL-12Rβ1 and IL-12Rβ2, the other heterodimeric cytokine IL-23 of the same family utilizes a heterodimer of IL-12Rβ1 and IL-23R for biological activities [6,8,48]. In other studies, we have also described the involvement of IL-12Rβ1 in biological functions of p40_2_ [9]. As expected, these receptors are internalized after successful binding with their respective ligands [5,6]. However, we have seen that p40 pretreatment is capable of reversing the effect of p40_2_, IL-12, and IL-23 and restoring the surface expression of IL-12Rβ1 in p40_2_-, IL-12-, and IL-23-treated T cells [5]. On the other hand, p40 remains unable to stop the internalization of either IL-12Rβ2 or IL-23R [5]. Accordingly, we have also seen that that p40 neutralization by p40 mAb stimulates the internalization of IL-12Rβ1 via caveolin to cause the death of prostate cancer cells via the IL-12–IFN-γ pathway [4]. Therefore, it is likely that p40 mAb is also increasing the level of IFNγ in TNBC tumor of PDX mice via stimulation of IL-12Rβ1 internalization and upregulation of the IL-12 signaling pathways. 

It is known that blood monocytes infiltrate into tumors to be ultimately differentiated into macrophages, known as tumor-associated macrophages (TAMs) [49,50]. TAMs can be divided into specific subsets based on marker, function, and phenotype.

For example, it is widely accepted that arginase 1-expressing and polyamine-producing tumor-associated M2 macrophages (TAM2) support pro-oncogenic functions and are pathogenic in cancer [50,51].

On the other hand, being characterized by the expression of inducible nitric oxide synthase (iNOS) and tumor necrosis factor α (TNFα), tumor-associated M1 macrophages (TAM1) are known to exhibit anti-cancer activity via proinflammatory immune responses [51]. Therefore, reprogramming immunosuppressive TAM2 towards a pro-inflammatory TAM1 state is known to limit tumor growth [52]. Accordingly, we have seen very few TAM1 and many TAM2 in TNBC tumors of untreated PDX mice. However, p40 mAb immunotherapy markedly upregulated TAM1 and downregulated TAM2 in TNBC tumors of PDX mice. It has been suggested that TAM2s develop under the influence of tumor microenvironment where TGFβ level is abundant and that the TAM-TGFβ crosstalk leads to tumor immune escape thereby stimulating the proliferation of tumor cells [53,54,55]. Therefore, for an immunotherapy to be successful in cancer treatment, it is important to overcome the resistance from TAM-TGFβ signaling. It is nice to see that consistent to the promotion of TAM1 and suppression of TAM2, p40 mAb treatment markedly suppressed the TGFβ axis in TNBC tumors. Therefore, p40 mAb immunotherapy is capable of suppressing different death-evading signaling pathways in TNBC tumors and future studies are warranted to delineate if IL-12Rβ1 plays any role in p40 mAb-mediated modulation of the TAM-TGFβ crosstalk.

## 5. Conclusions

In summary, here, we delineate upregulation of p40 in breast cancer patients, human TNBC cells and PDX mouse model of TNBC and demonstrate that scavenging of p40 by p40 mAb enriches anti-oncogenic CD4^+^IFNγ^+^ and CD8^+^IFNγ^+^ T cells, enriches M1 macrophages, downregulates M2 macrophages, suppresses TGFβ signaling, and induces apoptosis and/or necrosis, leading to tumor regression in a PDX mouse model of TNBC. Although the diverse disease process of human TNBC is not precisely the same as PDX mouse model of TNBC, our results suggest that p40 mAb may provide a new immunotherapeutic avenue against TNBC.

## Figures and Tables

**Table 1:** List of primers used in this study.

Gene	Directions	Sequence (5′…3′)
GAPDH	Sense	GCATCTTCTTGTGCAGTGCC
	Antisense	TACGGCCAAATCCGTTCACA
Cytochrome C	Sense	CCCCCAGCCTCCCTTATCTT
	Antisense	GGTCTGCCCTTTCTCCCTTC
Caspase 3	Sense	GAGCTTGGAACGGTACGCTA
	Antisense	CCGTACCAGAGCGAGATGAC
Caspase 8	Sense	AACATTCGGAGGCATTTCTGT
	Antisense	AGAAGAGCTGTAACCTGTGGC
Caspase 9	Sense	CTCTGAAGACCTGCAGTCCC
	Antisense	CTGCTCCACATTGCCCTACA
P53	Sense	ACCAGGGCAACTATGGCTTC
	Antisense	AGTGGATCCTGGGGATTGTG
BAD	Sense	CAGCGTACGCACACCTATCC
	Antisense	CGGGATCGGACTTCCTCAAG
BID	Sense	TCTGAGGTCAGCAACGGTTC
	Antisense	TTTGTCTTCCTCCGACAGGC
BAX	Sense	CTGGATCCAAGACCAGGGTG
	Antisense	CCTTTCCCCTTCCCCCATTC
BCL2	Sense	AGCATGCGACCTCTGTTTGA
	Antisense	GCCACACGTTTCTTGGCAAT
BCL-XL	Sense	TTGTACCTGCTTGCTGGTCG
	Antisense	CCCGGTTGCTCTGAGACATT
BAK	Sense	CCTGGGCCAACACGC
	Antisense	CTGTGGGCTGAAGCTGTTCTA

## Data Availability

Not applicable.
