# Correction: Kundu et al. Regression of Triple-Negative Breast Cancer in a Patient-Derived Xenograft Mouse Model by Monoclonal Antibodies against IL-12 p40 Monomer. *Cells* 2022, *11*, 259

**DOI:** 10.3390/cells13151297

**Published:** 2024-08-01

**Authors:** Madhuchhanda Kundu, Sumita Raha, Avik Roy, Kalipada Pahan

**Affiliations:** 1 Department of Neurological Sciences, Rush University Medical Center, Chicago, IL 60612, USA; madhuchhanda.kundu@gmail.com (M.K.); sumita_raha@rush.edu (S.R.); avik61332@gmail.com (A.R.); 2 Division of Research and Development, Jesse Brown Veterans Affairs Medical Center, 820 South Damen Avenue, Chicago, IL 60612, USA

**Error in Figure 3 and Figure 9**

In the original publication [1], there were mistakes in Figure 3 and Figure 9.

In Figure 3B, two panels of the IgG group overlapped. One of the images of the control group of Figure 3C also overlapped with another figure.

There was also a copy–paste error in Figure 9A (bottom two panels of the p40 mAb group). Instead of a magnified image on the last panel, we pasted the unmagnified one.

These are all honest mistakes that happened while preparing the figures in the PowerPoint. Please see the corrected Figures below.

**Figure 3:**
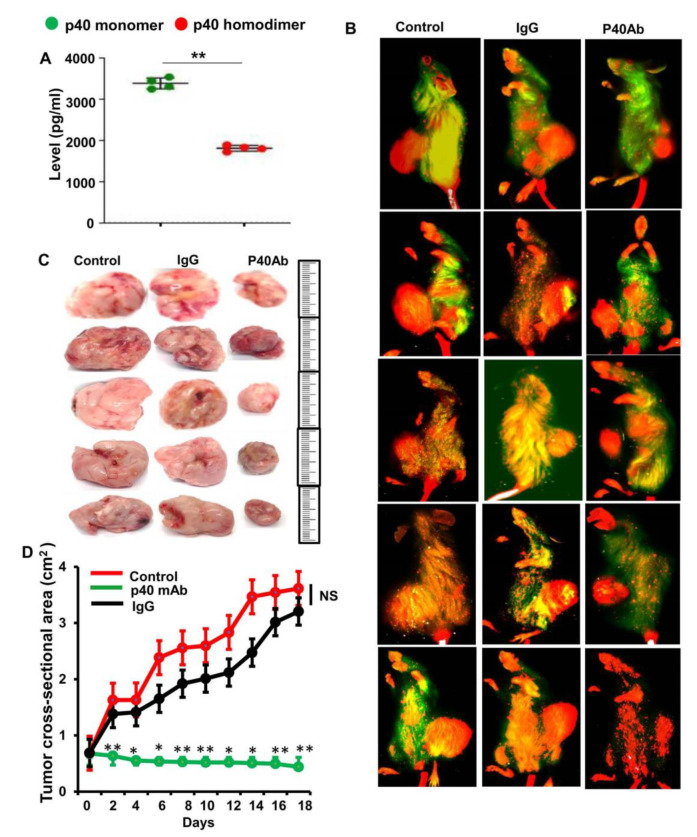
Regression of TNBC tumor in vivo in patient-derived xenograft (PDX) mice by p40 mAb. (**A**) Female 6–8 week old NOD scid gamma (NSG) mice were engrafted TNBC tumor fragments at passage P1-P9 (invasive ductal carcinoma; TNBC ER^−/−^/PR^−/−^/HER2^−/−^) in the flank. After 6 weeks of engraftment, levels of p40 and p40_2_ were measured in serum by sandwich ELISA. Results are mean ± SEM of four mice (*n* = 4) per group. ** *p* < 0.01. (**B**) After about 4 weeks of engraftment, when tumors of PDX mice (*n* = 5 per group) were 0.6–0.8 cm^2^ in size, mice were treated with p40 mAb (right panel) and hamster IgG (middle panel) at a dose of 2 mg/kg body wt once a week. After 2 weeks, tumors were labeled with Alexa800 conjugated 2DG dye via tail vein injection and then imaged in Licor Odyssey infrared imaging system. Results were compared with control group (Left panel). (**C**) Tumors were excised from the flank of all groups of mice. Five mice (*n* = 5) were included in each group. (**D**) Tumor size was monitored every alternate day. Results are mean ± SEM of five different mice.

**Figure 9:**
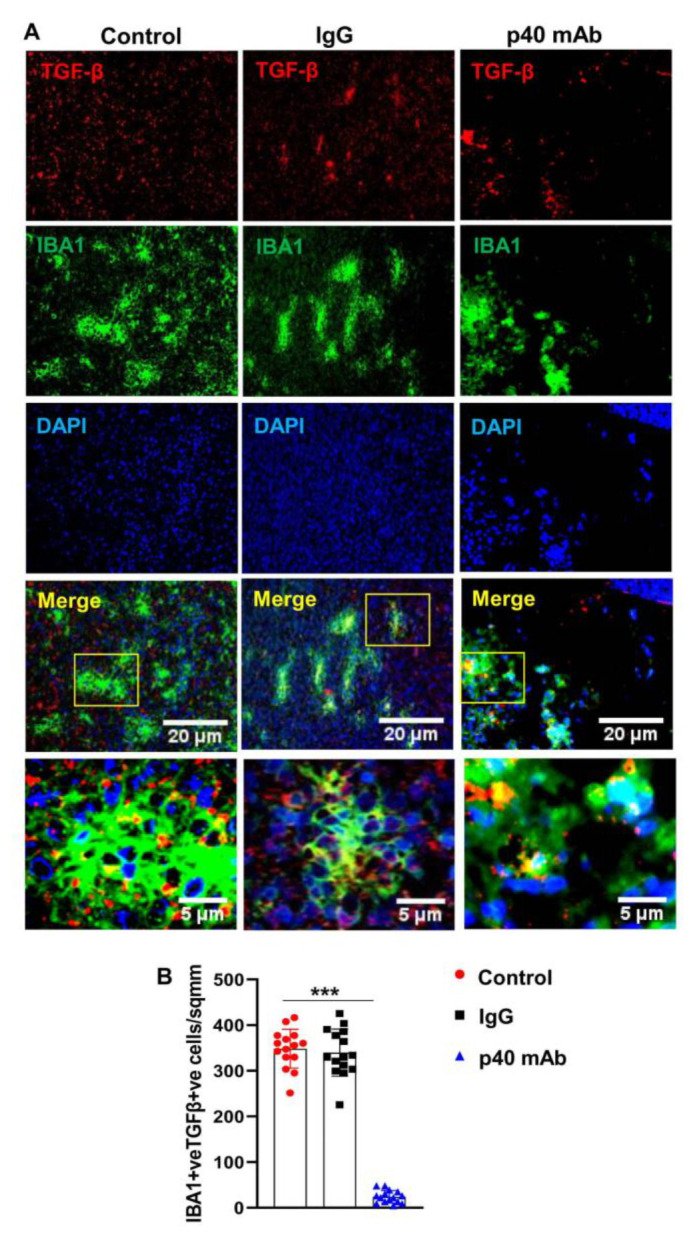
Neutralization of p40 by p40 mAb decreases the level of TGFβin TNBC tumor of PDX mice. Female 6–8 week old NSG mice were engrafted TNBC tumor in the flank. After about 4 weeks of engraftment, when tumors of PDX mice were 0.6–0.8 cm^2^ in size, mice were treated with p40 mAb and hamster IgG at a dose of 2 mg/kg body wt once a week. After 2 weeks of treatment, tumor cross sections were double-immunolabeled for Iba1 and TGFβ (**A**). DAPI was used to stain nuclei. Cells positive for Iba1 and TGFβ (**B**) were counted in one section (2–3 images per section) of each of five different mice per group. * *p* < 0.05; *** *p* < 0.001.

The authors state that the scientific conclusions are unaffected. This correction was approved by the Academic Editor. The original publication has also been updated.
